# Knowledge, Attitude and Practice of Tehran’s Inhabitants for an Earthquake and Related Determinants

**DOI:** 10.1371/4fbbbe1668eef

**Published:** 2012-08-06

**Authors:** Abbas Ostad Taghizadeh, Mostafa Hosseini, Iman Navidi, Ali Asghar Mahaki, Hassan Ammari, Ali Ardalan

## Abstract

Background A major destructive earthquake is predicted to shake the Tehran city in the near future. To mitigate the damage from such earthquakes, it is necessary to assess the preparedness of people and find the related risk factors. Methods A cross-sectional study was conducted in Tehran city among people aged 15 years or older in 2009. 1195 of Tehran's residents were interviewed using a questionnaire. Pearson chi-square test and binary logistic regression were used in order to evaluate the factors associated with preparedness against an earthquake. Results The analysis showed that 1076 (90.0%), 1160 (97.1%), and 490 (41.0%) of the participants achieved half of the possible scores for the knowledge, attitude, and practice components, respectively. Furthermore, in multivariate analysis low knowledge (p<0.001), having a high-school (p=0.033) or lower education (p<0.001) and living in Northern high-risk regions (p<0.001) of the Tehran were identified as risk factors for taking precautionary measures against earthquake. For low knowledge, lack of previous experience (p<0.001), and working as labor, businessman, employee (p=0.001) or being housewife (p=0.002) were related risk factors. In addition, people in the Southern high risk regions were significantly more knowledgeable (OR=0.618 compared to people in low risk regions) about earthquakes. Conclusions It is suggested that preparedness programs should target people with lower educational level and people in high risk regions especially the Northern districts of the city and aim at increasing public knowledge about earthquakes. Address for correspondence: Ali Ardalan, No. 78, Italia Ave, Department of Disaster Public Health, School of Public Health, Tehran University of Medical Sciences, Tehran, Iran. Email: aardalan@gmail.com or aardalan@tums.ac.ir Citation: Ostad Taghizadeh A, Hosseini M, Navidi I, Mahaki AA, Ammari H, Ardalan A. Knowledge, Attitude and Practice of Tehran’s Inhabitants for an Earthquake and Related Determinants. PLOS Currents Disasters. 2012 Aug 6

## Introduction

Based on the observations since 1900, one earthquake of magnitude 8 or greater, 15 earthquakes of magnitude 7-7.9 and 134 earthquakes of magnitude 6-6.9 on the Richter scale are expected each year, worldwide.^1^ The number of large earthquakes has remained relatively constant, however, due to the increasing number of seismological stations, the observed number of smaller earthquakes (of magnitude lesser than 6) has increased each year.^2^ During the first decade of the 21^st^ century, 4,022 natural disasters have been reported, 284 (7%) of which were earthquakes. Although they constitute a small share among the number of disasters, earthquakes are the major cause of death (55.7%) and cause US$232,070 million worth of damage (22%) comparing to other natural phenomena.^3^ Earthquakes have been the cause of 97.0% of deaths occurred as a result of natural hazards in Iran during the last four decades.

Tehran, the capital city and the most inhabited city of Iran, is located in a high seismic zone that is the result of its location in the main part of Alpine-Himalayan orogenic belt. The city has recorded many earthquakes during its history.^4^ Many studies have suggested that a large earthquake is expected to occur in the near-future.^5^ ^6^ Historical data from 4 BC to 1830 AD show that eight large destructive earthquakes shook this region and caused severe damages to Shahre Ray, a part of Tehran at the present time. More recently, three notable earthquakes that occurred in this region are those of Buin Zahra (1962 ), Changureh (Avaj, 2002 ) and Firozabad Kojor (2004 ).^7^ The 1962 Buin Zahra earthquake resulted in 12,225 fatalities.^8^

The Bam earthquake was the latest major earthquake in Iran, this shook the Bam city and a large area of the Kerman province on December 26, 2003.^9^ More than 30,000 people were killed, 30,000 injured, about 75,000 became homeless and 85% of buildings damaged or destroyed.^8^ The Bam earthquake can be considered a turning point in the improvement of Iran’s disaster risk reduction efforts at national and local levels.^10^

This disaster made the Iranians more cautious about the same event in the capital city of Tehran, to an extent that the Iranian government considered moving the capital to another city^11^ to reduce the population of this region which is estimated to be more than 12 million.^12^

To mitigate the damage from the earthquakes, some precautionary actions can be taken, such as earthquake insurance or fixing high furniture securely; however, people in both developing and developed countries do not utilize these actions.^13^

The question "Why some people become prepared for natural hazards but not all?" or in other words "What motivates people to take precautionary actions in order to mitigate damage from the hazards?" has been addressed abundantly in the literature. The answers are varied depending on the type of hazard and the region studied. The findings show that the income and economic level^14^ ^15^ , educational level^15^ ^16^ , home ownership^17^ ^18^ ^19^ and length of residence^20^ are factors associated with taking precautionary actions against various hazards in some regions. Some factors such as social class for flood preparedness are argued to be significant in some regions but not in others. The latest research which has some similarities in socioeconomic levels with Iran was conducted in Istanbul by Tekeli-Yeşil et al.^16^ Their findings suggested that educational level is the major factor associated with taking precautionary measures against an earthquake. Location of the home, participating in rescue actions after previous earthquakes, knowledge about earthquake, tenure, attitude score, age and general safety are other factors that were associated with taking precautionary measures against an earthquake.

Only a few researches have studied the factors associated with earthquake preparedness in Iran. As indicated above, the factors may vary between regions. The latest published research studied the factors using a KAP study with data from 2007.^12^ Their findings suggested that occupation, location of the home, age group and education are among the factors that can influence the knowledge, attitude and practice toward the earthquake preparedness.

After the Bam earthquake, the Iranian government has executed several preparedness programs. It is thus necessary to assess the efficiency of these programs and to identify the social groups that are in most need of related interventions. We also considered it necessary to reevaluate the population preparedness for earthquake and identify differences among different social groups. In addition, we aimed to estimate the associated factors using statistical methods.

## Methods

**Sample and Procedure.** A cross-sectional survey conducted by the Social and Cultural Studies Department, Tehran Municipality employed a questionnaire of 80 questions. This study focused on 31 of those questions, which included the participants' demographic characteristics, their knowledge about earthquakes and their attitude toward earthquakes and uptake of precautiounary measures.

The target population included residents of Tehran 15 years of age or older, staying in Tehran in the year of 2009. Initially, the sample size for the estimation of components of interest (Knowledge, Attitude and Practice regarding earthquake preparedness) was calculated using n=(z^2^_1-α/2_×p×(1-p))/d^2 ^with p=0.50 for each component considering α=0.05 and the precision of 3% (d=0.03) which yielded a total number of 1067 to be a reasonable for the aims of this study. However, using a random stratified-systematic sampling method, 1195 records were finally sampled.

First, in each of the 22 city's districts, the number of samples were calculated proportionally to the size of the district's population. Second, a block was chosen randomly in each district. Finally, trained interviewers started from the first unit of the block, filled the questionnaire for the household based on the responses from a person who was older than 15 years and capable to answer the questions. Then the interviewer systematically skipped next three units, interviewed the fifth household and continued until the end of the block. In the case that there were not enough samples in the block, interviewers filled the remaining questionnaires in the right hand side block.

**Measurements**

**Demographic variables.** Eight questions were asked by the interviewers to assess the demographic characteristics of the participants: age, sex, education, previous earthquake experience, home ownership, length of residence, marital status and occupation.

**Region.** Based on the district of each participant and the location of the faults which are showed in Fig. 1, the region of each participant was categorized into three groups: (I) Low risk regions: These districts are not on active seismic faults (districts #6,7,8,9,10,11,12,17,18). (II) Southern high-risk regions (districts #13,14,15,16,19,20) and (III) Northern high-risk regions (districts #1,2,3,4,5,21,22): The Southern and Northern districts of Tehran which were on active seismic faults. Distinguishing the Northern and Southern high-risk districts (regions) was due to different socio-economic characteristics of these regions. In addition, previous studies have shown that buildings in the Northern districts are less vulnerable to the earthquake than those in the Southern area.^6^

**Tehran Districts and Faults:**
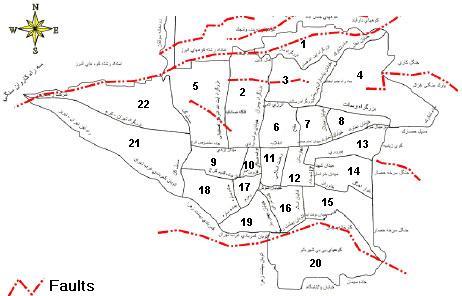
Source: Available online at http://abbasimehr.ir/blog/wp-content/uploads/2010/04/tehran_fault_map.jpg

**Knowledge about earthquake preparedness.** Ten questions were asked to evaluate the participants' knowledge of safety measures during an earthquake. Each question asked about safe places during an earthquake. Participants got 1 score for each correct answer and 0 for an incorrect one . The sum of the scores was used as the knowledge score for each participant. The median of the score of all participants was calculated and any participant with a score lower than the median, was regarded as a participant with low knowledge.

**Attitude toward earthquake preparedness.** Three questions were asked to assess the attitude of the participants toward the earthquake. Questions like "Earthquakes can be avoided by praying" were in this section. A score from 1 through 5 was set to each question in which 1 stands for the lowest attitude and 5 for the highest. The sum of these three questions was assumed as the attitude score (Cronbach's α=0.65). Any participant with an attitude score lower than median was regarded as participant with low attitude.

**Taking precautionary actions (Practice component).** Nine yes/no questions were asked by interviewers to evaluate the measure of precautionary actions for each participant. The questions were about the precautionary actions that can mitigate the damages of earthquakes such as "Does your house have an earthquake insurance?". The sum of yes answers considered as the practice score (Cronbach's α=0.74). The median for this score which considered as cut-off point was 3, thus any participant with practice score lower than 3 considered as participant with low practice.

**Analysis.** SPSS 11.5 have been used in order to record and analyze the data. All demographic variables were assumed as categorical variables. To avoid the effect of missing values, for each of knowledge, attitude and practice (KAP) components, the summation based on the mean of answered questions was assumed as the score. For example if three questions out of ten of the knowledge component were missing, the mean of the seven remained questions multiplied by 10 assigned as the score of knowledge for that observation.

The score of the KAP components were analyzed and median used as the cut off point for categorizing these components, such that if an observation's knowledge score was bellow the median, that observation was considered as having low knowledge. The same applied to the attitude and practice components and three new binary variables extracted.

We used frequency tables to present the characteristics of study population. As the "other" group in the marital status category and the "military" group in the occupation category had a small number of observations, we removed them from further analysis in this study. Cross-tabulation was used in order to present the distribution of the answers based on the factor variables for each component and chi-square test applied to test whether the components and demographic variable were independent or not. Instead of chi-square p-value, the p-value of Fisher Exact test is reported wherever it was possible to use this test.

To assess the relationship of demographic factors and KAP components by adjusting other factors, multiple binary logistic regression was used. For each regression model, the significance level and odd ratio and its 95% confidence interval are reported. Factors which were significant (at α≤0.2 level) at previous univariate tests entered in the regression model and the forward likelihood ratio method was used to dismiss the effect of non-significant factors. As low knowledge and attitude toward earthquake were significant factors for practice components, we entered these two component as well as other significant factors into the regression model for low practice. P-value < 0.05 was supposed as significant for all logistic regressions and 0.05 assigned as entry probability for the stepwise method.

## Results

Between June and October 2009, 1195 Tehran's residents were interviewed in Tehran, Iran. The characteristics of the study population are shown in Table I. Of the participants, 717 (60.1%) were aged 21-45 years old and the ratio of male to female was nearly 1. In addition, 322 (27.0%) had received education lower than high school or were or illiterate and about 30% of the participants were single. Furthermore, 959 (79.9%) of the participants had faced an earthquake at least once in the past and 1014 (90.9%) of them had been living in Tehran for more than ten years. Besides, 1076 (90.0%), 1160 (97.1%), and 490 (41.0%) achieved half of the possible scores for knowledge, attitude and practice components, respectively.

**Table I. Study Population Characteristics:** *The difference between total frequencies for each variable is due to the missing values. †Sum of percent column for each variable may not be 100 because of the decimal rounding.

Variable	Group	Frequency^*^	Percent^†^
Age group
	15-12	133	11.1%
	21-45	717	60.1%
	46-65	266	22.3%
	>65	78	6.5%
Sex
	Male	623	52.1%
	Female	572	47.9%
Educational level
	Illiterate	55	4.6%
	Under high school	267	22.4%
	High school	516	43.3%
	Higher educations	353	29.6%
Earthquake experience			
	Yes	949	79.9%
	No	238	20.1%
Home ownership
	Owner	808	68.7%
	Tenure	368	31.3%
Length of residence
	Less than 3 years	35	3.1%
	3 to 10 years	66	5.9%
	More than 10 years	1014	90.9%
Region
	Low risk region	438	36.7%
	Southern high risk region	335	28.0%
	Northern high risk region	422	35.3%
Marital status
	Single	353	36.7%
	Married	819	28.0%
	Other	16	35.3%
Occupation
	Labor, businessman, or employee	418	35.3%
	Student	238	20.1%
	Housewife	379	32.0%
	Jobless	47	4.0%
	Military	6	0.5%
	Retired	97	8.2%

Table II displays the results of univariate analysis of the factors affecting KAP components as well as the association between knowledge and attitude components with practice.

Results from Table II show that low knowledge about earthquake was significantly associated with higher age groups and lack of previous earthquake experience, and had a significant association with the occupation and region of the participant. Lower attitude was significantly associated with increasing age, length of residence and being married. The attitude component also had a significant negative association with educational level and was associated with sex, region and occupation of the participant. Furthermore, there was a significant association between the practice component and previous earthquake experience, region of the participant, low knowledge about earthquake and low attitude toward earthquake, and there was a significant negative relation between educational level and practice component. It should be noted that this results are tested independently; it means that the associations found here might be confounded by the effect of other factors.

**Table II. Univariate Analysis of Association between Factors and KAP Components Using Pearson Chi-square and Fisher Exact Tests:** * Fisher exact test

Factor	Group	Low knowledge		Low attitude		Low Practice	
		n (%)	p-value	n (%)	p-value	n (%)	p-value
Age group			0.036		<0.001		0.336
	15-20	40 (30.3%)		81 (61.4%)		59 (44.4%)	
	21-45	312 (43.6%)		358 (50.2%)		318 (44.4%)	
	46-65	114 (43.5%)		185 (72.5%)		123 (46.2%)	
	>65	34 (44.2%)		58 (76.3%)		43 (55.1%)	
Sex*			0.557		0.018		0.245
	Male	267 (43.0%)		334 (54.7%)		273 (43.9%)	
	Female	234 (41.3%)		348 (61.5%)		271 (47.4%)	
Educational level			0.171		<0.001		<0.001
	Illiterate	26 (48.1%)		43 (81.1%)		39 (70.9%)	
	Under high school	124 (46.4%)		171 (66.3%)		152 (56.0%)	
	High school	215 (42.2%)		312 (61.1%)		223 (43.3%)	
	Higher educations	135 (38.2%)		154 (43.9%)		128 (36.3%)	
Marital status*			0.561		<0.001		0.201
	Single	142 (40.5%)		171 (49.0%)		149 (42.2%)	
	Married	345 (42.4%)		496 (61.5%)		380 (46.5%)	
Occupation			0.040		0.001		0.108
	Labor, businessman, or employee	190 (45.6%)		217 (52.8%)		180 (43.2%)	
	Student	79 (33.3%)		122 (51.5%)		96 (40.3%)	
	Housewife	165 (43.9%)		246 (65.8%)		189 (49.9%)	
	Jobless	20 (42.6%)		27 (57.4%)		23 (48.9%)	
	Retired	41 (43.2%)		60 (65.2%)		49 (50.5%)	
Home ownership*			0.949		0.277		0.487
	Owner	340 (42.3%)		468 (59.1%)		374 (46.3%)	
	Tenure	154 (42.1%)		204 (55.6%)		162 (44.0%)	
Earthquake experience*			<0.001		>0.999		0.019
	Yes	363 (38.5%)		546 (58.0%)		414 (43.6%)	
	No	132 (55.5%)		133 (58.1%)		124 (52.3%)	
Length of residence			0.933		0.028		0.231
	Less than 3 years	13 (37.1%)		14 (40.0%)		20 (57.1%)	
	3 to 10 years	27 (40.9%)		36 (54.5%)		26 (39.4%)	
	More than 10 years	402 (39.9%)		610 (61.1%)		454 (44.8%)	
Region			0.004		0.006		0.011
	Low risk region	204 (46.9%)		254 (58.7%)		178 (40.7%)	
	Southern high risk region	117 (35.0%)		210 (64.0%)		151 (45.1%)	
	Northern high risk region	180 (43.0%)		218 (52.4%)		215 (50.9%)	
Low knowledge*			-		-		<0.001
	Yes	-		-		281 (56.1%)	
	No	-		-		259 (37.8%)	
Low attitude*			-		-		0.033
	Yes	-		-		325 (47.7%)	
	No	-		-		205 (41.4%)	

In order to adjust for the effect of possible confounders, a multiple binary logistic regression method was used. Table III shows the result of logistic regression of knowledge component on factors using forward method. The result of this analysis shows that lack of previous earthquake experience, region and occupation of the participants remained as significant predictors for low knowledge.

**Table III. Multivariate Logistic Regression Analysis of the Association between Factors and Low Knowledge:** † Only the factors that were significant in the last step are reported here * P < 0.01, ** P < 0.001

Factor^†^	Group	OR	95% CI	
Earthquake experience				
	Yes	1		
	No**	1.980	1.471	2.665
Region				
	Low risk region	1		
	Southern high risk region*	0.618	0.456	0.836
	Northern high risk region	0.864	0.655	1.141
Occupation				
	Student	1		
	Labor, businessman, or employee*	1.786	1.272	2.507
	Housewife*	1.732	1.223	2.451
	Jobless	1.773	0.925	3.399
	Retired	1.617	0.985	2.655

Older age and lower educational level remained as risk factors on low attitude toward earthquake (Table IV). In addition, low practice was significantly linked to lower levels of education, low knowledge and region of the participants when educational level, previous earthquake experience, region of the participant, low knowledge and low attitude were entered in a multivariate logistic regression, as shown in Table V.

**Table IV. Multivariate Logistic Regression Analysis of the Association between Factors and Low Attitude:** † Only the factors that were significant in the last step are reported here * P < 0.01, ** P < 0.001

Factor†	Group	OR	95% CI	
Age group				
	21-45	1		
	15-20	1.313	0.883	1.953
	45-65**	2.233	1.608	3.100
	> 65*	2.252	1.256	4.039
Educational level				
	Higher education	1		
	Illiterate*	3.363	1.573	7.188
	Under high school**	1.926	1.352	2.742
	High school**	1.806	1.352	2.411

**Table V. Multivariate Logistic Regression Analysis of the Association of Factors with Low Practice:** † Only the factors that were significant in the last step are reported here * P < 0.05, ** P < 0.001

Factor†	Group	OR	95% CI	
Educational level				
	Higher education	1		
	Illiterate**	4.825	2.517	9.251
	Under high school**	2.422	1.721	3.408
	High school*	1.365	1.025	1.819
Low knowledge				
	No	1		
	Yes**	2.110	1.656	2.688
Region				
	Low risk region	1		
	Southern high risk region	1.147	0.845	1.558
	Northern high risk region**	1.711	1.289	2.271

## Discussion

Our findings showed that people with a lower educational level are at higher risk of unpreparedness than people with a higher education. People in the Northern high-risk regions are less prepared than people in low-risk regions. The analysis also showed that low knowledge can cause low preparedness. Low knowledge, however, is affected by previous earthquake experience, the region and occupation by itself. Furthermore, the attitude component was also affected by the educational level and age group.

In the latest study conducted in Tehran^12^ , age, occupation and location of the participants were suggested to be associated with low preparedness. Although our results showed higher risk for older people, the difference was not significant in both univariate and multivariate analysis. Occupation had the same results in our study. Also, our study showed a significant difference in region groups which is similar to the study by Jahangiri *et al.* Our study showed that people in high-risk regions are less prepared than people in low risk regions. The reason for this might be the reliance of people in Northern vulnerable regions on their secure buildings^21^ or cultural aspects related to higher socio-economic class or less attention to the media.

In our study, the attitude toward earthquake was not a significant factor for taking precautionary actions against an earthquake after multivariate analysis, however, Tekeli‐Yeşil et al.^16^ showed that it was a significant factor together with education, location of the home, knowledge, previous earthquake experience, home ownership, age and general safety in Istanbul, Turkey. Our analysis showed that attitude and knowledge about an earthquake are significantly related to each other. As the relationship between knowledge and the practice component was also significant in our study, this might have resulted in the removal of attitude from the multivariate analysis.

As in other cities around the world, different regions of Tehran have different socio-economic characteristics . If we consider the region in our study as a socio-economic factor, our findings replicate the effect of economic level and social classes on the practice component which was shown in the USA^14^, Turkey^15^ , and Iran^22^ . However, our analysis did not show any significant association between home ownership and practice which was on the other hand reported in New Zealand^19^ and Turkey^16^,nor length of residence as shown in a study from the USA.^20^

Although we tried to assess the effect of all the factors used by previous studies, we did not have data on some of them, such as the risk perception which was employed previously.^16^ ^22^ Spittal *et al.* have distinguished the survival and mitigate types of preparedness 19 , however, in our study, the practice component was a combination of these two. Although we distinguished the Southern and Northern high-risk regions to check the effect of secure and insecure buildings, it is suggested to use building type as an independent factor for future research.

## Conclusions

Our findings show that general education, region and knowledge about earthquakes are the factors which can influence the behavior of Tehran residents during an earthquake. Therefore, in order to mitigate the damage from future earthquakes, it will be importnat to increase the population's general education as well as their knowledge about earthquakes, especially for those in high-risk regions.
